# Evaporation coefficient and condensation coefficient of vapor under high gas pressure conditions

**DOI:** 10.1038/s41598-020-64905-5

**Published:** 2020-05-18

**Authors:** Kotaro Ohashi, Kazumichi Kobayashi, Hiroyuki Fujii, Masao Watanabe

**Affiliations:** Division of Mechanical and Space Engineering, Faculty of Engineering, Hokkaido University, Sapporo, 060-8628 Japan

**Keywords:** Fluid dynamics, Phase transitions and critical phenomena, Physical chemistry

## Abstract

We investigated the evaporation and condensation coefficients of vapor, which represent evaporation and condensation rates of vapor molecules, under high gas pressure (high gas density) conditions in a system of a vapor/gas-liquid equilibrium state. The mixture gas is composed of condensable gas (vapor) and non-condensable gas (NC gas) molecules. We performed numerical simulations of vapor/gas–liquid equilibrium systems with the Enskog–Vlasov direct simulation Monte Carlo (EVDSMC) method. As a result of the simulations, we found that the evaporation and condensation fluxes decrease with increasing NC gas pressure, which leads to a decrease in the evaporation and condensation coefficients of vapor molecules. Especially, under extremely high gas pressure conditions, the values of these coefficients are close to zero, which means the vapor molecules cannot evaporate and condensate at the interface. Moreover, we found that the vapor molecules behave as NC gas molecules under high gas pressure conditions. We also discussed the reason why NC gas molecules interfere with evaporation and condensation of vapor molecules at the vapor/gas–liquid interface.

## Introduction

Evaporation and condensation are fundamental phenomena in the field of not only chemistry and physics^1^ but also in several applications^2–4^. Evaporation/condensation is a non-equilibrium phenomenon; hence, the Navier-Stokes equation system, which is based on macroscopic continuum mechanics, cannot predict the correct quantity of mass, momentum, and energy flux across the vapor/gas–liquid interface due to evaporation/condensation.

Vapor flows accompanied with evaporation and condensation have been studied based on the molecular gas dynamics analysis using the Boltzmann equation. Important information of vapor flows with evaporation and condensation has been obtained from previous studies^5,6^. The kinetic boundary condition (KBC), which is the boundary condition at the gas–liquid interface, is needed to solve the Boltzmann equation. The KBC contains parameters called the evaporation coefficient and condensation coefficient, which represent the rate of evaporating and condensing molecules at the vapor-liquid interface^7–9^. These parameters cannot be determined in the framework of the analysis of the Boltzmann equation; hence, several studies were conducted to determine the values of evaporation and condensation coefficients for a single-component vapor–liquid system using molecular dynamics^10–15^ and the kinetic theory of gases^16–19^.

In reality, evaporation and condensation occur in a multi-component system. For example, droplet evaporation under high ambient pressure surrounded by multi-component gases has been widely studied for fuel combustion^20^. Nucleation of small water droplets due to condensation in the atmosphere also has been studied for meteorology^21^. Moreover, the collapse of a spherical bubble filled with condensable gas (vapor) and non-condensable gas (NC gas) has been analyzed^22–24^. One of the present authors pointed out that, in the final stage of the bubble collapse, extremely high-concentration of NC gas is formed at the liquid surface^23^. It was hypothesized that this high-concentration NC gas prevents evaporation and condensation of vapor molecules in the final stage of bubble collapse. However, this hypothesis has not been clarified yet.

The detailed mechanism of evaporating/condensing molecular behaviors has been discussed for single-component systems^9,25,26^. On the other hand, there are few pieces of research concerning KBC which targets a multi-component system, e.g., vapor–NC gas mixture problem. The authors conducted MD simulations of vapor–NC gas binary mixtures using the Ar-Ne system in equilibrium^27^. However, because it was necessary to treat not only vapor molecules but also the NC gas molecules in MD simulation, it was difficult to discuss the influence of NC gas molecules on evaporation and condensation behaviors of vapor molecules and to deal with a very high NC gas density (high NC gas pressure) state. Hence, the behavior of vapor and NC gas molecules in several pressure conditions at the interface has not been completely understood yet.

In this study, we investigated the evaporation and condensation coefficients of vapor when the NC gas is at a high pressure in the systems. No study has investigated the evaporation and condensation of vapor with high-pressure NC gas on the microscopic molecular level. We focus on the Enskog-Vlasov equation^28^ as the expression of the vapor/gas-liquid systems. The Enskog–Vlasov direct simulation Monte Carlo (EVDSMC) method proposed by Frezzotti *et al*.^29–31^, which is a numerical method to solve the Enskog–Vlasov equation, is an advantage to MD simulation because the EVDSMC method runs 100 times faster with the same particle number^32^. We investigated the value of evaporation and condensation coefficients of vapor in a high-pressure vapor/gas-liquid equilibrium with EVDSMC method in the present study.

## Definitions of Evaporation Coefficient and Condensation Coefficient

**Figure 1:**
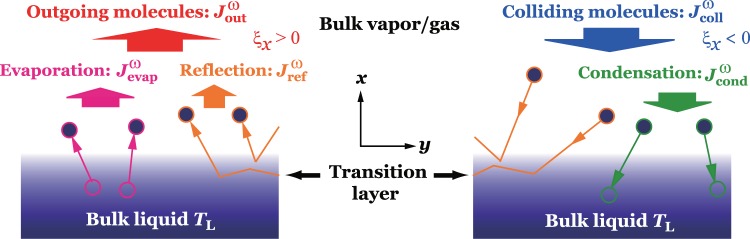
Molecular mass fluxes at a vapor/gas–liquid interface. ${J}_{{\rm{call}}}^{\omega }$ denotes the colliding molecular mass flux, ${J}_{{\rm{out}}}^{\omega }$ is the outgoing molecular mass flux, ${J}_{{\rm{evap}}}^{\omega }$ is the evaporating molecular mass flux, ${J}_{{\rm{cond}}}^{\omega }$ is the condensing molecular mass flux, and ${J}_{{\rm{ref}}}^{\omega }$ is the reflecting molecular mass flux. $\omega =V$ denotes the molecular mass fluxes for vapor molecules at the interface, and $\omega =G$ those for NC gas molecules.

Figure 1 shows a schematic of molecular mass fluxes at a vapor/gas-liquid interface. The superscript $\omega =(V,G)$ indicates vapor (*V*) or NC gas (*G*) as shown in the following; ${J}_{{\rm{coll}}}^{\omega }$ is the colliding molecular mass flux composed of molecules come from the vapor/gas phase and collide onto the liquid phase. ${J}_{{\rm{out}}}^{\omega }$ is the outgoing molecular mass flux composed of molecules come from the liquid phase and go out to the vapor/gas phase. ${J}_{{\rm{evap}}}^{\omega }$ is the evaporating molecular mass flux, that is a part of ${J}_{{\rm{out}}}^{\omega }$, composed of molecules evaporate into the gas/vapor phase from the liquid phase. ${J}_{{\rm{cond}}}^{\omega }$ is the condensing molecular mass flux, that is a part of ${J}_{{\rm{coll}}}^{\omega }$, composed of molecules condensate to the liquid phase. ${J}_{{\rm{ref}}}^{\omega }$ is the molecular mass flux, that is a part of ${J}_{{\rm{out}}}^{\omega }$ and ${J}_{{\rm{coll}}}^{\omega }$, composed of molecules reflect on the interface and go to the vapor/gas phase. The relationships between these molecular mass fluxes are expressed as

1 $${J}_{{\rm{out}}}^{V}={J}_{{\rm{evap}}}^{V}+{J}_{{\rm{ref}}}^{V},\,{J}_{{\rm{coll}}}^{V}={J}_{{\rm{ref}}}^{V}+{J}_{{\rm{cond}}}^{V},\,{J}_{{\rm{out}}}^{G}={J}_{{\rm{evap}}}^{G}+{J}_{{\rm{ref}}}^{G},\,{J}_{{\rm{coll}}}^{G}={J}_{{\rm{ref}}}^{G}+{J}_{{\rm{cond}}}^{G}\mathrm{}.$$

Here evaporation coefficient ${\alpha }_{e}^{\omega }$ and condensation coefficient ${\alpha }_{c}^{\omega }$ for binary gas mixture are defined as follows^27^:

2 $${\alpha }_{e}^{V}=\frac{{J}_{{\rm{evap}}}^{V}}{{J}_{{\rm{out}}}^{V\ast }},\,{\alpha }_{c}^{V}=\frac{{J}_{{\rm{cond}}}^{V}}{{J}_{{\rm{coll}}}^{V}},\,{\alpha }_{e}^{G}=\frac{{J}_{{\rm{evap}}}^{G}}{{J}_{{\rm{out}}}^{G\ast }},\,{\alpha }_{c}^{G}=\frac{{J}_{{\rm{cond}}}^{G}}{{J}_{{\rm{coll}}}^{G}},$$

where superscript * means the value of the vapor/gas–liquid equilibrium state. The evaporation coefficient is defined as the ratio of the evaporating molecular mass flux to the outgoing molecular mass in an equilibrium state, and the condensation coefficient is the ratio of condensing molecular mass flux to the colliding molecular mass flux regardless of the equilibrium or non-equilibrium state. NC gas molecules cannot evaporate and condensate at the interface; NC gas molecules dissolve into or degas from liquid instead. However, for convenience to treat the coefficients, we call ${\alpha }_{e}^{G}$ and ${\alpha }_{c}^{G}$ as the evaporation and condensation coefficients for NC gas molecules.

The KBC ${f}_{{\rm{out}}}^{\omega }$, which is the velocity distribution function (VDF) of molecules, includes these coefficients as follows^12,16,27^:

3 $${f}_{{\rm{out}}}^{V}=\frac{{\alpha }_{e}^{V}{n}^{V\ast }+\mathrm{(1}-{\alpha }_{c}^{V}){n}_{{\rm{ref}}}^{V}}{{\mathrm{(2}\pi {R}^{V}{T}_{L})}^{\mathrm{3/2}}}\exp \left(-\frac{{\xi }_{x}^{2}+{\xi }_{y}^{2}+{\xi }_{z}^{2}}{2{R}^{V}{T}_{L}}\right),\,{f}_{{\rm{out}}}^{G}=\frac{{\alpha }_{e}^{G}{n}^{G\ast }+\mathrm{(1}-{\alpha }_{c}^{G}){n}_{{\rm{ref}}}^{G}}{{\mathrm{(2}\pi {R}^{G}{T}_{L})}^{\mathrm{3/2}}}\exp \left(-\frac{{\xi }_{x}^{2}+{\xi }_{y}^{2}+{\xi }_{z}^{2}}{2{R}^{G}{T}_{L}}\right),({\xi }_{x} > 0),$$

where ${n}^{V\ast }$ is the saturated vapor number density that is the function of liquid temperature ${T}_{L}$, and ${n}^{G\ast }$ is the number density of NC gas molecules at equilibrium under specified NC gas pressure and temperature. In this condition, there is also NC gas dissolution depending on the NC gas pressure. ${R}^{\omega }$ is the gas constant for $\omega$ component molecule, $\xi =({\xi }_{x},{\xi }_{y},{\xi }_{z})$ is the molecular velocity, and the subscripts denote the direction. *x* indicates the normal direction to the interface, and *y, z* indicate the tangential directions to the interface. ${n}_{{\rm{ref}}}^{\omega }$ is the number density composed of reflecting molecules at the vapor/gas-liquid interface and is represented as

4 $${n}_{{\rm{ref}}}^{V}=-\,\sqrt{\frac{2\pi }{{R}^{V}{T}_{L}}}{\int }_{-\infty }^{\infty }{\int }_{-\infty }^{\infty }{\int }_{-\infty }^{0}{\xi }_{x}\,{f}_{{\rm{coll}}}^{V}d{\xi }_{x}d{\xi }_{y}d{\xi }_{z},\,{n}_{{\rm{ref}}}^{G}=-\,\sqrt{\frac{2\pi }{{R}^{G}{T}_{L}}}{\int }_{-\infty }^{\infty }{\int }_{-\infty }^{\infty }{\int }_{-\infty }^{0}{\xi }_{x}\,{f}_{{\rm{coll}}}^{G}d{\xi }_{x}d{\xi }_{y}d{\xi }_{z},$$

where ${f}_{{\rm{coll}}}^{\omega }$ is the VDF that comes from the vapor/gas phase and collides onto the liquid phase; this VDF is obtained by solving the Boltzmann equation. In a equilibrium state, ${n}_{{\rm{ref}}}^{V}={n}^{V\ast }$ and ${n}_{{\rm{ref}}}^{G}={n}^{G\ast }$. In this study, ${\alpha }_{e}^{\omega }$ and ${\alpha }_{c}^{\omega }$ are organized by *μ*, which is a molar fraction of dissolved NC gas, at a liquid temperature. *μ* is defined as *μ* = ${n}^{G{\rm{liq}}}/({n}^{V{\rm{liq}}}+{n}^{G{\rm{liq}}})$, where ${n}^{\omega {\rm{liq}}}$ denotes the number density in the bulk liquid phase closest to the interface. Hence, we define the KBC as the function of bulk liquid temperature and molar fraction. This definition of KBC is same with the previous study^27^.

### Simulation method

The EVDSMC method proposed by Frezzotti *et al*.^29–31^ is based on the Enskog–Vlasov equation^28^; therefore, we show the Enskog–Vlasov equation briefly. The Enskog–Vlasov equation is an approximate kinetic equation that can describe vapor/gas-liquid two-phase flow. The validity of the equation for evaporation/condensation problem was discussed and the application for the evaporation/condensation problem have been conducted^16,30,31,33^.

In this simulation, we assume the molecular masses of vapor and NC gas, ${m}^{V}$ and ${m}^{G}$, take same value ${m}^{V}={m}^{G}=m$, and these diameters, ${\sigma }^{V}$ and ${\sigma }^{G}$, also take the same value ${\sigma }^{V}={\sigma }^{G}=\sigma$ for the ease of analysis.

The Enskog-Vlasov equation assumes that molecules interact by the Sutherland potential as follows:

5 $${\psi }^{VV}=(\begin{array}{cc}+\infty  & (r\le \sigma )\\ -{\varphi }^{VV}{\left(\frac{\sigma }{r}\right)}^{\gamma } & (r > \sigma ),\end{array}\,{\psi }^{GG}=(\begin{array}{cc}+\infty  & (r\le \sigma )\\ -{\varphi }^{GG}{\left(\frac{\sigma }{r}\right)}^{\gamma } & (r > \sigma ),\end{array}\,{\psi }^{VG}={\psi }^{GV}=(\begin{array}{cc}+\infty  & (r\le \sigma )\\ -{\varphi }^{VG}{\left(\frac{\sigma }{r}\right)}^{\gamma } & (r > \sigma ),\end{array}$$

where $\psi$ represents the intermolecular potential, and φ and *γ* are constants that relate with potential well. The superscript means interaction between the components; namely, *VV* represents the interaction between vapor and vapor, *GG* represents that between NC gas and NC gas, and *VG* and *GV* represent that between vapor and NC gas. In this simulation, we set $\gamma =6$, and *r* is the distance between the molecules.

The Enskog–Vlasov equation governs VDF, $f({\bf{x}},\xi ,t)$. ${\bf{x}}=(x,y,z)$ is the position, $\xi =({\xi }_{x},{\xi }_{y},{\xi }_{z})$ is the molecular velocity, and *t* is the time. $f({\bf{x}},\xi ,t)d{\bf{x}}d\xi$ denotes the number of molecules in the small 6-dimensional box, $d{\bf{x}}d\xi$. The Enskog–Vlasov equations for the VDFs of vapor and NC gas are written as

6 $$\begin{array}{ccc}\frac{\partial {f}^{V}}{\partial t}+\xi \cdot \frac{\partial {f}^{V}}{\partial {\bf{x}}}+\frac{{{\bf{F}}}^{V}}{m}\cdot \frac{\partial {f}^{V}}{\partial \xi } & = & {C}_{E}({f}^{V},{f}^{V})+{C}_{E}({f}^{V},{f}^{G}),\\ \frac{\partial {f}^{G}}{\partial t}+\xi \cdot \frac{\partial {f}^{G}}{\partial {\bf{x}}}+\frac{{{\bf{F}}}^{G}}{m}\cdot \frac{\partial {f}^{G}}{\partial \xi } & = & {C}_{E}({f}^{G},{f}^{G})+{C}_{E}({f}^{G},{f}^{V}).\end{array}$$

**Figure 2:**
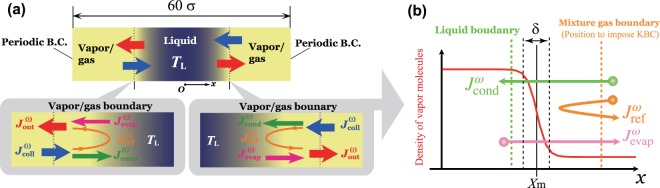
(**a**) Schematic of the present simulation condition (steady vapor/gas–liquid equilibrium). At the center of the system, there is a thin liquid film; (**b**) Schematic of the molecular fluxes in the vicinity of vapor/gas–liquid interface. We can count the number of molecules using mixture gas and liquid boundaries; ${J}_{{\rm{evap}}}^{\omega }$ denotes the evaporating molecular mass flux for $\omega$ component molecules, ${J}_{{\rm{ref}}}^{\omega }$ the reflecting molecular mass flux, and ${J}_{{\rm{cond}}}^{\omega }$ the condensing molecular mass flux.

*C*_*E*_ in the above equations is the so-called collision term and it changes VDF by molecular collision:

7 $$\begin{array}{ccc}{C}_{E}({f}^{i},{f}^{j}) & = & {\sigma }^{2}{\int }_{-{\rm{\infty }}}^{{\rm{\infty }}}{\int }_{-{\rm{\infty }}}^{{\rm{\infty }}}{\int }_{-{\rm{\infty }}}^{{\rm{\infty }}}{\int }_{0}^{4\pi }\{Y\left[n,({\bf{x}}+\frac{\sigma \hat{{\bf{k}}}}{2})\right]\,{f}^{j}({\bf{x}}+\sigma \hat{{\bf{k}}},{\xi }_{1}^{\ast },t){f}^{i}({\bf{x}},{\xi }^{\ast },t)-Y\left[n,({\bf{x}}-\frac{\sigma \hat{k}}{2})\right]\,{f}^{j}({\bf{x}}-\sigma \hat{{\bf{k}}},{\xi }_{1},t){f}^{i}({\bf{x}},\xi ,t)\}H({\bf{g}}\cdot \hat{{\bf{k}}})({\bf{g}}\cdot \hat{{\bf{k}}}){d}^{2}\hat{{\bf{k}}}d{\xi }_{1},\end{array}$$

where *H* is the Heaviside step function, and **g** is the relative molecular velocities. ${\xi }^{\ast }$ and ${\xi }_{1}^{\ast }$ are the post-collision molecular velocities which relates the pre-collision molecular velocity *ξ* and *ξ*_1_ with unit vector $\hat{{\bf{k}}}$. *Y* is the pair correlation function in statistical mechanics^34^ and the function of number density as follows: *Y*(*n*) = $\frac{1}{2}\frac{2-\eta }{{\mathrm{(1}-\eta )}^{3}}$, and *η* = $\frac{\pi {\sigma }^{3}n}{6}$. The increase in pair correlation function is related to the molecular collision frequency with the increase of the number density at the colliding position. ${{\bf{F}}}^{\omega }=({F}_{x}^{\omega },{F}_{y}^{\omega },{F}_{z}^{\omega })$ is a so-called mean-field force and represents the approximated attractive intermolecular force. The detailed explanation of the equations is written in the previous paper^31^. In this study, we solve the Enskog–Vlasov equation with the EVDSMC method numerically. The EVDSMC method is an extension of the direct simulation Monte Carlo (DSMC) method^35^. From the simulation, we can get the macroscopic quantities of molecules. We consider a spatially one-dimensional system, and its width is $[\,-\,30\sigma \mathrm{,30}\sigma ]$. Eight simulations run with different numbers of sample NC gas molecules, as shown in Table 1. Figure 2(a) shows the schematic configuration of this simulation. In this simulation, we set ${\varphi }^{VV}=1.325k{T}_{0}$, ${\varphi }^{VG}=0.364k{T}_{0}$, and ${\varphi }^{GG}=0.1k{T}_{0}$, where *T*_0_ is the critical temperature of vapor expressed as $k{T}_{0}=0.094329\frac{4\gamma }{\gamma -3}{\varphi }^{VV}$. As an initial condition, we set vapor sample molecules as a liquid in the region of $[\,-\,10\sigma \mathrm{,10}\sigma ]$ uniformly. In another region, we set NC gas sample molecules uniformly. We impose the periodic boundary condition at $x=\,\pm 30\sigma$. The thermostat algorithm controls the liquid temperature^30,36^ at ${T}_{L}=0.633{T}_{0}$. After reaching the equilibrium state, we take the sampling to get the macroscopic variables from the sample molecules as shown in Table 1.

**Table 1:** Number of sample molecules for each simulation case; *N*_0_ is the number of reference molecules, *N*^*V*^ is the number of sample vapor molecules, *N*^*G*^ is the number of sample NC gas molecules, and *N*_*S*_ is the number of samples, where *N*_*S*_ = 4000 in this study.

Case No.	*N*_0_	*N*^*V*^ × *N*_*s*_	*N*^*G*^ × *N*_*s*_
1	500	500,000,000	25,000,000
2	500	500,000,000	65,000,000
3	500	500,000,000	125,000,000
4	500	500,000,000	250,000,000
5	500	500,000,000	500,000,000
6	500	500,000,000	625,000,000
7	500	500,000,000	750,000,000
8	500	500,000,000	1,000,000,000

To calculate evaporation and condensation coefficients, we count the number of evaporating, reflecting, and condensing molecules across the vapor/gas–liquid interface. The counting method in this simulation was proposed by one of the authors^17^ referring to the previous studies^11,37^. Using this method, the value of evaporating mass flux agrees with that of the spontaneous evaporating mass flux obtained by the vacuum evaporation simulation^12^.

Using mixture gas and liquid boundaries shown in Fig. 2(b), we can determine the molecular fluxes for each component. The position of the mixture gas boundary is the position to impose KBC. To locate the boundaries, the following equation fits the number density distribution:

8 $${n}^{V}(x)=\frac{{n}^{Vgas}+{n}^{V{\rm{liq}}}}{2}+\frac{{n}^{Vgas}-{n}^{V{\rm{liq}}}}{2}\,\tanh \left(\frac{x-{X}_{m}}{0.455\delta }\right),$$

where ${n}^{Vgas}$ is the number density of vapor in bulk vapor/gas phase, *δ* is the so-called 10–90 thickness, which denotes the thickness of the transition layer, and *X*_*m*_ is the center of the 10–90 thickness. The positions of mixture gas boundary *x*_*gb*_ and liquid boundary *x*_*lb*_ are defined as follows by referring to the previous study^27^: ${x}_{gb}={X}_{m}+3\delta$, ${x}_{lb}={X}_{m}-\delta$.

Using the following procedure, we count the molecular mass fluxes across the vapor/gas–liquid interface: 1. ${J}_{{\rm{evap}}}^{\omega }$ is counted from the evaporating molecules that pass the liquid boundary at *x*_*lb*_ and then the mixture gas boundary at *x*_*gb*_; 2. ${J}_{{\rm{ref}}}^{\omega }$ is counted from the reflecting molecules that pass the mixture gas boundary twice at *x*_*gb*_ without passing the liquid boundary; 3. ${J}_{{\rm{cond}}}^{\omega }$ is counted from the condensing molecules that pass the mixture gas boundary at *x*_*gb*_ and then the liquid boundary at *x*_*lb*_.

From the above procedures, the non–dimensional molecular mass fluxes are obtained as

9 $$\hat{J}=\frac{J{\sigma }^{3}}{m\sqrt{2R{T}_{0}}}=\frac{N}{{N}_{0}}\frac{\sigma }{\Delta t\sqrt{2R{T}_{0}}}\frac{\Delta x}{\sigma }{n}_{c}^{V}{\sigma }^{3},$$

where $R=k/m=k/{m}^{V}=k/{m}^{G}$, *k* is the Boltzmann constant, *N* is the number of sample molecules that pass the arbitrary position within time $\Delta t$, ${N}_{0}/{V}_{{\rm{cell}}}$ is the number of reference smaple molecules which relates to the critical number density ${n}_{c}^{V}$ in each cell, and ${V}_{{\rm{cell}}}$ is the volume of cell. $\Delta x$ is the length of cell in *x* direction. We set $\Delta \hat{x}=\Delta x/\sigma =0.2$ and $\Delta \hat{t}=\Delta t/(\sigma /\sqrt{2R{T}_{0}})=0.0005$ in the present study.

## Results and discussion

### Macroscopic quantities and Henry’s law for NC gas

**Figure 3:**
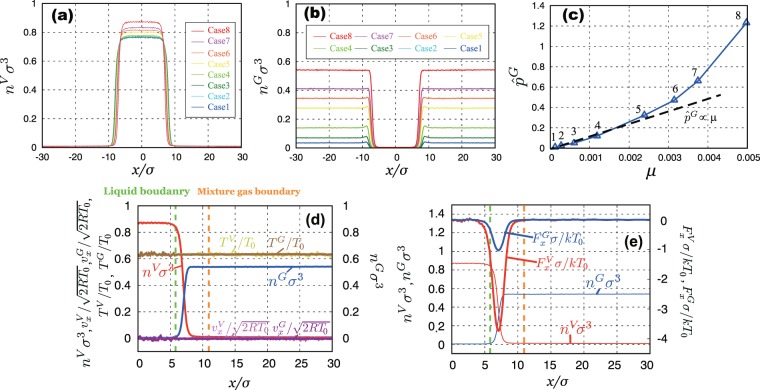
Number density profiles of each simulation case: (**a**) vapor component, ${n}^{V}{\sigma }^{3}$; (**b**) NC gas component, ${n}^{G}{\sigma }^{3}$; (**c**) non-dimensional NC gas pressure ${\hat{p}}^{G}={p}^{G}{\sigma }^{3}/k{T}_{0}$ as a function of molar fraction of dissolved NC gas molecules *μ*. Each number near the symbols denotes each case number. The dotted line shows the eye guide (linear relation of the Henry’s law). Macroscopic quantities of Case 8: (**d**) number density, velocity, and temperature fields; (**e**) mean-field forces of vapor and NC gas molecules.

Figure 3(a,b) show the number density distributions of vapor and NC gas components obtained by the equilibrium simulations. In Fig. 3(a), the liquid phase composed of vapor molecules located between about $x/\sigma =-\,7.5$ and $x/\sigma =7.5$. As shown in Fig. 3(a), the value of the density of the liquid phase for Case 1 is the lowest and that for Case 8 is the highest; the value of the NC gas phase of Case 1 is the lowest and that for Case 8 is the highest (Fig. 3(b)). These results indicate that the liquid density of vapor molecules increases as the NC gas density increases because of the high NC gas pressure.

Here, we confirm whether NC gas pressure satisfies Henry’s law. As stated above, the NC gas pressure increases as the number of NC gas molecules in the system increases. According to the law, the NC gas pressure increases linearly as the molar fraction of dissolved NC gas, *μ*, increases. The Enskog-Vlasov equation has three types of stress tensor^28^ composed of the kinetics, collision, and mean-field effect. We can calculate the partial pressure of each component using the definitions of stress tensor for the *xx*-component as follows:

$${p}^{\omega }={p}_{xx}^{(k)\omega }+{p}_{xx}^{(c)\omega }+\frac{1}{3}{p}_{xx}^{(t)\omega },$$

where _*pxx*_^(*k*)*ω*^ is the kinetic part, _*pxx*_^(*k*)*ω*^ is the collisional part, and 1/3_*pxx*_^(*k*)*ω*^ is the mean-field part.

Figure 3(c) shows the relation between *μ* and non–dimensional NC gas pressure ${\hat{p}}^{G}={p}^{G}{\sigma }^{3}/(k{T}_{0})$ calculated from the simulation. Each number in Fig. 3(c) denotes each case number. The dotted line shows the eye guide (linear relation of the Henry’s law). As shown in the figure, we can see that the value of *μ* increases with the case number. The simulation almost satisfies the Henry’s law about up to $\mu =0.002$. In contrast, in the case of $\mu  > 0.002$, the value deviates from Henry’s law; the very high gas pressure condition is realized in this simulation. In the present simulation, the non–dimensional saturated vapor pressure at liquid temperature is about 0.004. In Case 8, the non–dimensional pressure of NC gas is 1.230. Hence, the NC gas pressure is about 310 times larger than the saturated vapor pressure. Replacing this with a water-air system at room temperature, the NC gas pressure is equivalent to over 10 atm. It cannot be quantitatively compared with the water-air system because of the difference in the molecules, but it can be said that the NC gas pressure becomes extremely high. In Cases 5–8, the value of the number density of vapor/gas phase cannot be ignored for that of liquids (see Fig. 3(b)); one cannot consider as the ideal gas. As the results, we suppose that the relation shown in Fig. 3(c) at high *μ* value does not satisfy the Henry’s law.

Figure 3(d) shows macroscopic quantities (number density, velocity, and temperature) of Case 8. We also show the mixture gas boundary and liquid boundary as described in the previous section. We can determine the molecular mass fluxes using theses boundaries. As mentioned above, NC gas density is the highest in Case 8. From the figure, we can see that the velocities of vapor and NC gas, ${v}_{x}^{V}$ and ${v}_{x}^{G}$, take uniformly zero, and the temperatures of vapor and NC gas, ${T}^{V}$ and ${T}^{G}$, also take uniformly constant value, ${T}^{V}={T}^{G}={T}_{L}=0.633{T}_{0}$ (controlled liquid temperature) in the whole region of the system. From the results, we conclude that the system reaches the vapor/gas–liquid equilibrium state.

Figure 3(e) shows the mean–field force (attractive force). Vapor molecules composing the liquid phase attract the NC gas molecules in the vicinity of the interface by its attractive force. As the results, the adsorption film is formed at the interface. This tendency also is same with the previous MD simulation^27^. As the attractive force becomes larger, we confirmed the existence of the adsorption film of NC gas molecules at the interface.

### Evaporation coefficient and condensation coefficient

**Figure 4:**
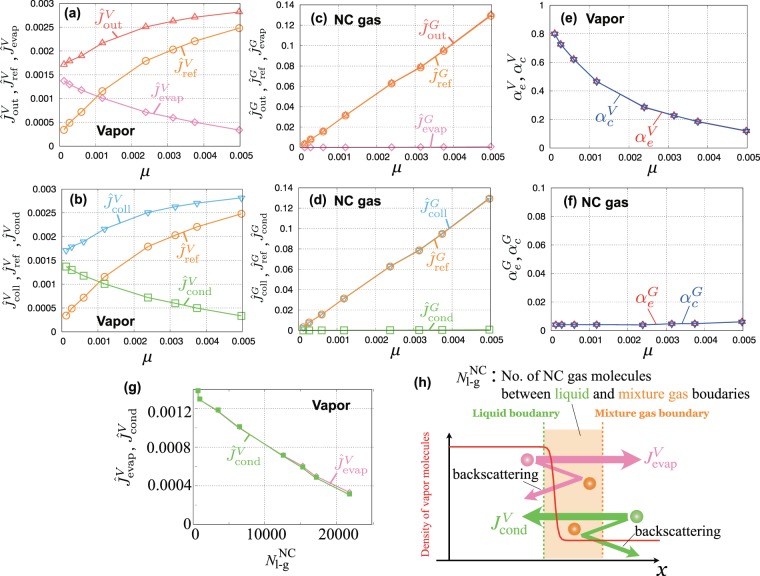
Molecular mass fluxes, evaporation coefficient, and condensation coefficient of vapor and NC gas molecules as a function of *μ*: (**a**) outgoing, evaporating, and reflecting vapor molecules; (**b**) colliding, condensing, and reflecting vapor molecules; (**c**) outgoing, evaporating, and reflecting NC gas molecules; (**d**) colliding, condensing, and reflecting NC gas molecules; (**e**) evaporation and condensation coefficients of vapor molecules; (**f)** evaporation and condensation coefficients of NC gas molecules; (**g**) evaporating and condensing molecular mass fluxes as the function of ${N}_{{\rm{l}}-{\rm{g}}}^{{\rm{NC}}}$; and (**h**) schematic of ${J}_{{\rm{evap}}}^{V}$ and ${J}_{{\rm{cond}}}^{V}$. Due to the collision of molecules between the liquid and mixture gas boundaries, the backscattering for ${J}_{{\rm{evap}}}^{V}$ and ${J}_{{\rm{cond}}}^{V}$ increases.

In this section, we show the molecular mass fluxes using the method described in the previous section and calculated evaporation and condensation coefficients by Eq. (2). Figure 4(a,b) show the outgoing and colliding vapor molecular mass fluxes at the position of mixture gas boundary (position to impose KBC), respectively. Figure 4(c,d) are those of NC gas molecules. They are organized by a molar fraction of dissolved NC gas *μ*.

For the mass fluxes of vapor molecules (Fig. 4(a,b)), we can see that ${J}_{{\rm{evap}}}^{V}$ and ${J}_{{\rm{cond}}}^{V}$ take same values and decrease with *μ*. In the equilibrium condition, the relation ${J}_{{\rm{evap}}}^{V}={J}_{{\rm{cond}}}^{V}$ should hold. Hence, we can also confirm that the present simulations reach equilibrium. In contrast, ${J}_{{\rm{ref}}}^{V}$ becomes larger as the value of *μ* increases. As a result, the outgoing and colliding fluxes, ${J}_{{\rm{out}}}^{V}(={J}_{{\rm{evap}}}^{V}+{J}_{{\rm{ref}}}^{V})$ and ${J}_{{\rm{c}}{\rm{o}}{\rm{l}}{\rm{l}}}^{V}(={J}_{{\rm{c}}{\rm{o}}{\rm{n}}{\rm{d}}}^{V}+{J}_{{\rm{r}}{\rm{e}}{\rm{f}}}^{V})$ and, take higher values with increasing *μ*.

For the mass fluxes of NC gas molecules (Fig. 4(c,d)), ${J}_{{\rm{ref}}}^{G}$ increases almost linearly as *μ* increases. This is because of the increase in number of NC gas molecules in the system. ${J}_{{\rm{evap}}}^{G}$ and ${J}_{{\rm{cond}}}^{G}$ take very lower values regardless of the value of *μ* because these molecules are non-condensable; the values of ${J}_{{\rm{out}}}^{G}$ and ${J}_{{\rm{coll}}}^{G}$ are almost equal to those of ${J}_{{\rm{ref}}}^{G}$.

Evaporation and condensation coefficients of vapor and NC gas are determined by these molecular mass fluxes as shown in the following. Figure 4(e) shows evaporation and condensation coefficients of vapor. When *μ* is relatively small, the density and pressure of NC gas are also relatively small (see Fig. 3(c) for NC gas pressure). From Fig. 4(e), we can see that the evaporation and condensation coefficients take same values and decrease with increasing *μ*; theses coefficients take the same value from the definitions in equilibrium. The linearly decreasing trend of evaporation and condensation coefficients in small *μ* value agrees with previous MD results^27^. Moreover, the computational efficiency of EVDSMC enables us to investigate the evaporation and condensation coefficients of vapor when NC gas density and pressure become higher over the condition in which Henry’s law is satisfied. In extremely high-pressure conditions, these coefficients become close to zero, which indicates that the evaporation/condensation is restricted at high-pressure conditions.

To investigate the decrease in evaporation and condensation coefficients, we show the evaporating and condensing mass fluxes as a function of the number of NC gas molecules between the mixture gas and liquid boundaries, ${N}_{{\rm{l}}-{\rm{g}}}^{{\rm{NC}}}$, as shown in Fig. 4(g). The definition of ${N}_{{\rm{l}}-{\rm{g}}}^{{\rm{NC}}}$ is shown in Fig. 4(h). From Fig. 4(g), the evaporating and condensing molecular mass fluxes linearly decrease with increasing number of NC gas molecules inside the mixture gas and liquid boundaries. The molecular collision frequency is proportional to the number density of molecules^35^; then, the evaporating and condensing molecular mass fluxes decrease due to the increase of backscattering mass fluxes because of the collision between the NC gas molecules inside the mixture gas and liquid boundaries (shown in Fig. 4(h)); as a result, the evaporation and condensation coefficients decrease.

Figure 4(f) shows evaporation and condensation coefficients of NC gas. These values are nearly zero (the order is $O{(10}^{-3})$) in spite of the difference of *μ* because NC gas does not evaporate and condensate. This result of NC gas molecules also agrees with the previous MD result^27^. In the following section, we show how the vapor molecular behavior changes due to the influence of the number of NC gas molecules at the interface.

### Velocity distribution function (VDF) of vapor or NC gas molecules

**Figure 5:**
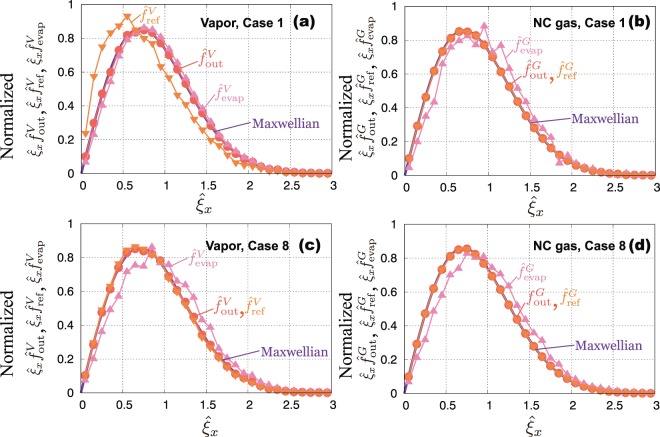
Molecular velocity distribution function (VDF) normalized to unity at the mixture gas boundary (the position to impose KBC): (**a**) vapor of Case 1; (**b**) NC gas of Case 1; (**c**) vapor of Case 8; and (**d**) NC gas of Case 8.

To investigate the molecular behavior at the interface, we show the molecular velocity distribution function (VDF) for outgoing, reflecting, and evaporating molecules through the interface at the mixture gas boundary. $\hat{f}=f{\sigma }^{3}{\mathrm{(2}R{T}_{L})}^{\mathrm{3/2}}$ represents the normalized molecular VDF for the molecular velocity of *x* direction normal to the interface, ${\hat{\xi }}_{x}={\xi }_{x}/\sqrt{2R{T}_{L}}$. Figure 5(a,b) show the results of Case 1 for the vapor and NC gas molecules, respectively. Figure 5(c,d) are the results of Case 8. The relation between ${\hat{f}}_{{\rm{out}}}^{\omega }$, ${\hat{f}}_{{\rm{evap}}}^{\omega }$, and ${\hat{f}}_{{\rm{ref}}}^{\omega }$ in the present situation is as follows:

10 $${\hat{f}}_{{\rm{out}}}^{\omega }={\alpha }_{e}^{\omega }{\hat{f}}_{{\rm{evap}}}^{\omega }+(1-{\alpha }_{c}^{\omega }){\hat{f}}_{{\rm{ref}}}^{\omega }$$

In Case 1(Fig. 5(a,b)), the VDFs of ${\hat{f}}_{{\rm{out}}}^{V}$ and ${\hat{f}}_{{\rm{out}}}^{G}$ are the Maxwellian at the liquid temperature. From the results, we can also confirm that the system reaches the equilibrium state. ${\hat{f}}_{{\rm{evap}}}^{V}$ and ${\hat{f}}_{{\rm{ref}}}^{V}$ slightly deviate from the Maxwellian: the mean speed of reflecting molecules becomes slower and that of evaporating molecules becomes slightly faster. This tendency is same with the previous MD simulations for single-component system^17^. On the other hand, for NC gas molecules, ${\hat{f}}_{{\rm{ref}}}^{G}$ takes almost Maxwellian; ${\hat{f}}_{{\rm{evap}}}^{G}$ slightly deviates from the Maxwellian. In the case of NC gas molecules, the values of ${\alpha }_{e}^{G}$ and ${\alpha }_{c}^{G}$ take $O{(10}^{-3})$ shown in Fig. 4(f). The deviation of ${\hat{f}}_{{\rm{evap}}}^{G}$ from the Maxwellian is not dominant for the total outgoing molecules ${\hat{f}}_{{\rm{out}}}^{G}$.

In Case 8(Fig. 5(c,d)), all of VDFs, ${\hat{f}}_{{\rm{out}}}^{V}$, ${\hat{f}}_{{\rm{ref}}}^{V}$, ${\hat{f}}_{{\rm{out}}}^{G}$, and ${\hat{f}}_{{\rm{ref}}}^{G}$, are almost same with the Maxwellian. On the contrary, ${\hat{f}}_{{\rm{evap}}}^{V}$ and ${\hat{f}}_{{\rm{evap}}}^{G}$ slightly deviate from the Maxwellian; the shapes of deviation are very similar between vapor and NC gas molecules, and also ${\hat{f}}_{{\rm{evap}}}^{G}$ of Case 1(see Fig. 5(b)). These results indicate that vapor molecules behave like NC gas when the gas pressure become high; as a result, outgoing, reflecting, and evaporating molecules take the same tendency for each component. We also confirmed that the VDFs for tangential component (*y* and *z* directions) take the Maxwellian at liquid temperature.

### Reflection of molecules at interface

**Figure 6:**
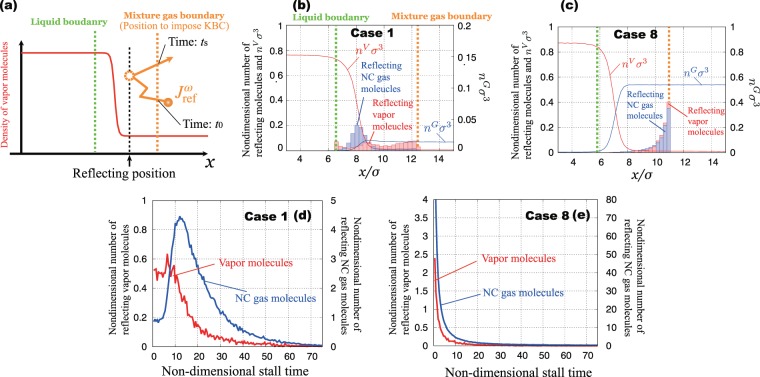
Non-dimensional number of reflecting molecules at the interface: (**a**) definition of reflect position of reflecting molecules between mixture gas and liquid boundaries; (**b**) non-dimensional number of reflecting molecules in Case 1; (**c**) non-dimensional number of reflecting molecules in Case 8; (**d**) non-dimensional stall time of reflecting for vapor and NC gas molecules in Case 1; (**e**) non-dimensional stall time of reflecting vapor and NC gas molecules in Case 8.

To investigate the reflection behavior of vapor and NC gas molecules, we show the reflecting position and staying time of vapor and NC gas molecules at the interface. We define the reflecting position of molecules at which the molecules reach the nearest position for the liquid boundary inside the region between the mixture gas and liquid boundaries (see Fig. 6(a)).

Figure 6(b) shows the reflecting position of Case 1. The red bar graph denotes the non-dimensional number of vapor molecules reflecting at the position, and the blue bar graph shows that of NC gas molecules. Vapor reflecting position toward vapor/gas phase covers all regions between the mixture gas and liquid boundaries. In contrast, NC gas reflects near the liquid boundary.

Figure 6(c) shows the reflecting position of Case 8. The reflecting position of vapor and NC gas toward the vapor/gas phase is almost the mixture gas boundary; the molecules coming from the vapor/gas phase collide with the high-density NC gas molecules inside the boundaries. Therefore, they are reflected toward the vapor/gas phase on mixture gas boundary immediately. This result also indicates that vapor behaves like NC gas in the condition that density and pressure of NC gas are very high.

We also measure the stall time of the reflecting molecules toward the vapor/gas phase. The stall time is defined as the staying time of reflecting molecules in the region between the mixture gas and liquid boundaries (see Fig. 6(a)). Figure 6(d,e) show non-dimensional stall time inside the boundaries versus non-dimensional number of reflecting molecules. Figure 6(d) shows the stall time of Case 1, and Fig. 6(e) shows that of Case 8. In Case 1, the NC gas molecules stay longer than the vapor molecules; the mean stall time of NC gas molecules is longer than that of vapor molecules. This is because of the attractive forces of vapor molecules in the vicinity of the interface. In contrast, in Case 8, each stall time of both molecules is shorter and reflects immediately in the vicinity of the mixture gas boundary. This result also suggests the vapor molecules behave like NC gas under high-pressure conditions.

The above results and discussion suggest that vapor does not evaporate and condensate in the high-pressure NC gas conditions. In this case, the evaporation coefficient ${\alpha }_{e}^{V}$ and condensation coefficient ${\alpha }_{c}^{V}$ are close to zero; Eq. (3) approaches the diffusion boundary condition. We also confirmed the influence of the value of intermolecular potential on the evaporation and condensation coefficients. Even if the different ${\varphi }^{VG}$, these coefficients decrease in the case of high gas pressure; the decreasing mechanism does not change. In future work, we will conduct non-equilibrium simulations of the vapor/gas–liquid binary system with EVDSMC method to investigate the values of the condensation and evaporation coefficients in the non-equilibrium states.

## Conclusions

We investigated the influence of high-pressure (high-density) NC gas on evaporation and condensation processes of vapor molecules in a vapor/gas–liquid equilibrium system using the EVDSMC method. The evaporating and condensing molecular mass fluxes of vapor molecules decrease with increasing NC gas molecules. As a result, the evaporation and condensation coefficients of vapor approach zero; evaporation and condensation are suppressed under high-pressure conditions.

We investigated the molecular behaviors in the vicinity of vapor/gas–liquid interface and found the following results:

1. Under low-pressure conditions, the velocity distribution functions of evaporating and reflecting molecules for vapor and NC gas take different tendency. In contrast, under high-pressure conditions, velocity distribution functions of vapor and NC gas molecules take the same tendency.
2. In low-pressure conditions, the NC gas molecules are more reflected in the vicinity of liquid boundary than the vapor molecules. In contrast, under high-pressure conditions, both the vapor and NC gas molecules reflect immediately in the vicinity of mixture gas boundary.
3. Under low-pressure conditions, the mean stall time of NC gas molecules takes longer than that of vapor molecules. In contrast, under high-pressure conditions, each stall time takes shorter and the same tendency.

These three results indicate that on the microscopic molecular level, the vapor molecules behave like NC gas molecules, that is, it is difficult for vapor molecules to condensate onto or evaporate from the liquid under high-pressure conditions.
