# Author Correction: Evaporation coefficient and condensation coefficient of vapor under high gas pressure conditions

**DOI:** 10.1038/s41598-020-68283-w

**Published:** 2020-07-01

**Authors:** Kotaro Ohashi, Kazumichi Kobayashi, Hiroyuki Fujii, Masao Watanabe

**Affiliations:** Division of Mechanical and Space Engineering, Faculty of Engineering, Hokkaido University, Sapporo, 060-8628 Japan

Correction to: *Scientific Reports* 10.1038/s41598-020-64905-5, published online 18 May 2020

This Article contains an error in the Results and Discussion section under the subheading ‘Macroscopic quantities and Henry’s law for NC gas’ where,

‘where _*pxx*_^(*k*)*ω*^ is the kinetic part, _*pxx*_^(*k*)*ω*^ is the collisional part, and 1/3_*pxx*_^(*k*)*ω*^ is the mean-field part.’

should read:

‘where ${p}_{xx}^{(k)\omega}$ is the kinetic part, ${p}_{xx}^{(c)\omega}$ is the collisional part, and ${1/3p}_{xx}^{(t)\omega}$ is the mean-field part.’

